# Microfluidic magnetic detection system combined with a DNA framework-mediated immune-sandwich assay for rapid and sensitive detection of tumor-derived exosomes

**DOI:** 10.1038/s41378-023-00617-w

**Published:** 2023-11-07

**Authors:** Qiuling Qian, Yutong Wei, Yi Xu, Mengmeng Zheng, Chenguang Wang, Shulin Zhang, Xiaoming Xie, Chaofeng Ye, Xianqiang Mi

**Affiliations:** 1 National Key Laboratory of Materials for Integrated Circuits, Shanghai Institute of Microsystem and Information Technology, Chinese Academy of Sciences, 865 Changning Road, Shanghai, 200050 China; 2 Shanghai Advanced Research Institute, Chinese Academy of Sciences, Shanghai, 201210 China; 3 University of Chinese Academy of Sciences, Beijing, 100049 China; 4 School of Information Science and Technology, Shanghai Tech University, Shanghai, 201210 China; 5 School of Life Sciences, Shanghai University, Shanghai, 200444 China; 6 School of Physics and Optoelectronic Engineering Hangzhou Institute for Advanced Study, University of Chinese Academy of Sciences, Chinese Academy of Sciences, Hangzhou, 310024 China

**Keywords:** Microfluidics, Biosensors

## Abstract

Tumor-derived circulating exosomes (TDEs) are being pursued as informative and noninvasive biomarkers. However, quantitatively detecting TDEs is still challenging. Herein, we constructed a DNA tetrahedral-structured probe (TSP)-mediated microfluidic magnetic detection system (μFMS) to provide a rapid and sensitive platform for analyzing TDEs. CD63 aptamer-modified Fe_3_O_4_ magnetic nanoparticles (MNPs) were constructed to form magnetic nano-report probes (MNRs). The microfluidic chips were fabricated from glass functionalized with DNA TSP-modified aldehyde groups and a PDMS layer designed with serpentine microchannels. An induction coil-based magnetic detector was used to measure the magnetic signal. The linear dynamic range of the μFMS system for TDE assays was 1.98 × 10^3^–1.98 × 10^7^ particles/mL with a limit of detection of 1.98 × 10^3^ particles/mL in PBS. There was no significant difference in TDE detection between the simulated serum and PBS, which indicated the feasibility of the constructed μFMS system for TDE analysis in complex biological systems. In terms of cost, reaction time and operation procedure, this μFMS has the potential to be developed as a clinical point-of-care testing tool for cancer diagnosis and therapeutics.

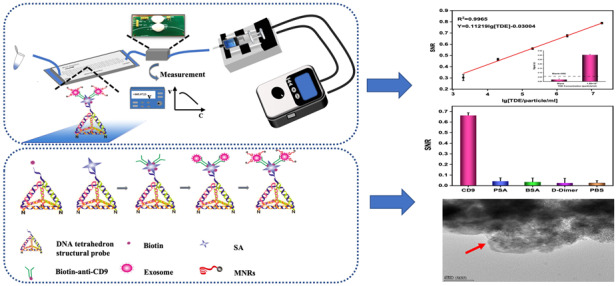

## Introduction

Cancer is currently the leading cause of death in 112 countries^1^ and seriously endangers human health. Exosomes are cup-shaped lipid bilayer membrane vesicles (30–150 nm in diameter) secreted by most cancer cells that contain bioactive lipids^2^, nucleic acids^3^, and protein cargo^4,5^. Tumor-derived circulating exosomes (TDEs) are released through outward blebbing of the cancer cell membrane^6–8^ and can mediate cell-cell communication by transmitting different signaling molecules, participating in a wide range of physiological and pathological processes in cancer^9,10^. An increasing number of studies have demonstrated that there are many TDEs in tissue and serum^11^ that could be recognized as cancer biomarkers for early diagnosis^12^. A variety of membrane proteins, such as membrane trafficking proteins (Rabs, Annexins), heat-shock proteins (Hsp 90, Hsp 70, Hsp60, and Hsc70) and tetraspanins (CD82, CD9, CD63, CD81), have been found on the surface of TDEs^5^. Among them, tetraspanins such as CD9 and CD63 have been used as exosome-specific markers for the identification and analysis of TDEs^13,14^.

To date, many TDE detection methods have been developed by recognizing and binding tetraspanins on the exosome surface, including fluorescence^15^, colorimetry^16,17^, ELISA^18^, surface plasmon resonance (SPR)^19^, surface-enhanced Raman scattering (SERS)^20–22^ and electrochemistry^23–25^. However, these methods have not been clinically used due to shortcomings such as relatively low detection performance, complicated operation and high cost. Therefore, it is essential to develop a sensitive, rapid and automatic detection platform for TDE analysis.

Recently, magnetic biosensors have emerged as powerful diagnostic platforms for molecular analysis, measuring targets including DNA, soluble proteins, exosomes and cells, with the advantages of intrinsically low background noise and stable signals in biological media^26^. Generally, magnetic nanomaterials (MNPs), labeling strategies and magnetometers are the key factors in magnetic biosensors for molecular analysis. MNPs, including ferrite MNPs, Fe-core MNPs and multicore MNPs, are attractive materials for sensing applications because of their unique magnetic properties and facile surface modification. Among them, ferrite MNPs have been more widely used for magnetic biosensors due to their high *M*_sat_ (saturation magnetization) values^27^, low toxicity, etc. Particle size is one of the factors that could affect the detection performance of magnetic biosensors, and MNPs with hydrodynamic diameters less than 50 nm have been demonstrated to be the ideal size^28,29^. The common labeling strategies for magnetic biosensors include clustering assays, sandwich assays and direct labeling. Sandwich magnetic labeling is one of the most widely used methods that employs affinity ligand-functionalized MNPs to capture biological targets. The affinity ligands couple with secondary affinity ligands fixed on a solid substrate surface to bring the MNPs to the sensor surface for magnetic signal detection. Affinity ligands such as aptamers, which can recognize targets with high affinity and specificity, have been increasingly used for conjugation with MNPs^30^. Various types of magnetometers have been applied to measure the magnetic signals produced by biological targets labeled with affinity ligand-functionalized MNPs, including fluxgate sensors^26^, superconducting quantum interference devices (SQUIDs)^31^, optically pumped atomic magnetometers (OPMs), magnetoresistive (MR) sensors^32,33^, Hall effect magnetometers^34^, and induction coils^35^. SQUID and OPM sensors have high detection sensitivity, but their potential for miniaturization and decreased cost is limited. Fluxgate sensors, magnetoresistive sensors and Hall sensors have been used for POCT detection^36^, but they have limited detection sensitivity. Induction coil-based magnetic detectors have the advantages of easy miniaturization, integration and low cost^36^. Combining induction coils with differential circuits could greatly reduce noise and improve the sensitivity of the magnetic detector. For instance, a magnetic sensor array made of induction coils and differential circuits has enabled the spatial imaging of magnetic nanoparticles with high sensitivity^35^.

Microfluidic chips can integrate a laboratory into a single chip to achieve rapid and automatic detection, making it possible to detect and analyze TDEs by offering an attractive combination of high throughput and sensitivity with low reagent consumption^37^. The constructed microfluidic chip for TDE detection usually consists of an upper cover and an underlying substrate^18,38,39^. To enhance capture efficiency, microstructures are often designed in the upper layer channel, providing a large surface area. The lower layer is generally used as a solid substrate to immobilize affinity ligands, such as antibodies and aptamers^18,39^.

Recently, self-assembly DNA nanotechnology has attracted much attention due to its extraordinary biostability and biocompatibility. Three-dimensional DNA TSPs have been introduced for the detection of DNA, microRNAs, CTCs and TDEs^40,41^. DNA TSPs can be easily immobilized on solid substrates by binding covalently with excellent controllability and highly precise orientations, which could reduce nonspecific adsorption and modulate the distribution and orientation of affinity ligands, such as antibodies and aptamers, thus increasing the detection sensitivity.

In this work, we developed a microfluidic magnetic detection system **(**μFMS) for the rapid and sensitive detection of TDEs by incorporating MNPs, microfluidic chips and induction coil-based magnetic detectors together with DNA TSPs. The CD63 aptamer-modified MNPs can transform the event of capturing TDEs into a magnetic signal with output in the form of a voltage signal. Induction coils with differential amplification circuits were utilized to eliminate most of the background noise. DNA TSPs were introduced and immobilized on aldehyde-modified glass slides (Fig. S3), acting as scaffolds to form capture structures for TDEs. The PDMS upper layer with a serpentine microchannel was tightly bonded with glass slides and integrated with the magnetic detection device. The μFMS was investigated and optimized for the automatic, sensitive and fast analysis of TDEs extracted from U251 cell lines.

## Experimental methods

### Reagents and materials

The DNA sequences (Table S1) were synthesized and purified by Sangon Biotech (Shanghai) Co. Ltd. Streptavidin (SA) was purchased from Sigma‒Aldrich (St. Louis, MO, USA). Biotinylated anti-PSA monoclonal antibody, biotinylated anti-BSA monoclonal antibody and biotinylated anti-D-dimer monoclonal antibody were purchased from Shanghai Linc-Bioscience Co. Ltd. Bovine serum albumin (BSA), Tween 20, and other chemicals were purchased from Sinopharm Chemical Reagent Co. Ltd. U251 cell lines were obtained from the Cell Bank of Shanghai Institute of Cell Biology, Chinese Academy of Sciences. Cell culture medium (DMEM), fetal bovine serum (FBS) and penicillin‒streptomycin (PS) were purchased from Invitrogen.

The DOWSIL^TM^ 184 polydimethylsiloxane (PDMS) prepolymer and curing agent were purchased from Dow Corning. The activation buffer for MNRs synthesis was MES (0.1 M, pH 6.0) containing 0.5 M NaCl; the coupling buffer was phosphate buffered saline (PBS) solution (100 mM sodium phosphate, 150 mM NaCl, pH 7.2); the western blot blocking buffer was TBST buffer (pH 7.5) containing 0.05% Tween-20; the TBE buffer was electrophoresis buffer containing 12.5 mM MgCl_2_; the TM buffer was Tris buffer (20 mM, pH 8.0) containing 50 mM MgCl_2_; and the TE buffer was DNA dissolving solution (10 mM Tris, 1 mM EDTA, pH 8.0). Milli-Q water (18 MΩ.cm^−1^ resistivity) was used throughout all experiments.

The dynamic light scattering (DLS) device was from Wyatt, DynaPro NanoStar. Transmission electron microscopy (TEM) was performed using FEI Tecnai Spirit G2 Bio TWIN. The polyacrylamide gel electrophoresis device and imaging device used in Western blot (WB) analysis were Bio-Rad, PowerPac Basic and Tanon, 1600, respectively. The nanoparticle tracking analysis (NTA) device consisted of a 405 nm laser, and the sCMOS camera was from Zeta View Ltd., Germany.

A polytetrafluoroethylene (PTFE) tube was purchased from Wenhao, Suzhou, Co., Ltd. A microsyringe pump was purchased from Longer pump, Boading, China. A Nano Drop Lite spectrophotometer was purchased from Thermo Fisher Scientific, USA. An O_2_ plasma cleaner (YZD08-2C) was purchased from SAOT (Beijing) Tech Co.

### Cell culture and TDE extraction

U251 cells were cultured in Dulbecco’s modified essential medium (DMEM, Shanghai, China) at 37 °C in a 5% CO_2_ humidified incubator. The culture medium contained 10% FBS and 1% 100 μg mL^−1^ streptomycin.

A standard ultracentrifugation method was used to obtain the TDEs. First, 140 mL of cell culture medium was transferred from the culture dish to 50 mL centrifuge tubes and centrifuged (1400 rpm, 10 min). Then, the supernatant was collected and centrifuged twice (3400 rpm, 20 min and 10,000 × g, 4 °C, 30 min). Second, the supernatant was collected, filtered through a pore filter (0.22 μm, Millipore), and centrifuged again (120,000 × g, 4 °C, 70 min). Then, the supernatant was removed, and the precipitate was resuspended in sterile PBS solution and ultracentrifuged (120,000 × g, 4 °C, 70 min). Finally, the supernatant was removed, and the precipitate (the obtained TDEs) was resuspended in sterile PBS solution (100 µL), divided into several Eppendorf tubes and stored at −80 °C.

### Characterization of extracted TDEs

TDEs secreted by U251 cell lines were characterized by TEM. Freshly extracted TDEs were diluted to 1.98 × 10^5^ particle/mL and dropped on a copper net for 20 min. After the floating liquid was removed, uranyl acetate was dropped on the copper net and mixed with TDEs. After negative dyeing at room temperature for 5 min, the copper net was washed with distilled water several times and observed by TEM.

To characterize the size distribution and measure the average size of the monodisperse population of isolated TDEs, 10 μL of extracted TDE sample was slowly taken to the bottom of the disposable cuvette at 25 °C and measured by DLS (30 sets of data each time for three cycles within five seconds).

To confirm that CD63 and CD9 were expressed on the surface of TDEs, the extracted TDEs were mixed with loading buffer (99 °C, 5 min) and separated by sodium dodecyl sulfate‒polyacrylamide gel electrophoresis (SDS‒PAGE). After the proteins were transferred from SDS‒PAGE gels to polyvinylidene difluoride (PVDF) membranes and incubated with milk for 1 h, primary antibodies against CD9 and CD63 were added and shaken slowly overnight at 4 °C. Then, the secondary antibody was added and incubated for 2 h at room temperature.

To analyze the size distribution and concentration of TDEs. TDEs were diluted in PBS to the optimal concentration and illuminated by the NTA laser beam. The typical Brownian motion of exosomes was observed.

### Preparation and characterization of DNA TSPs and MNRs

Five single-stranded DNAs, including one tetra-A strand, three amino-modified single DNA strands (tetra-B, tetra-C, and tetra-D) and one linker, were dissolved in TE buffer at a final concentration of 100 μM. One microliter of the single strand was mixed in 45 μL of TM buffer, and the mixed solution was heated to 95 °C for 10 min and cooled to 4 °C for 30 s using a T100^TM^ PCR Thermal Cycler. The synthesized DNA TSPs with a final concentration of 1 μM were confirmed by 8% PAGE and characterized by AFM.

MNRs were synthesized according to published procedures^42^. Twenty microliters of surface carboxyl-modified MNPs (2 mg/mL) was washed with 100 μL of MES buffer (pH=6.0) three times and then activated with EDC (100 μL, 2 mM)/NHS (100 μL, 5 mM) dissolved in 20 μL of MES buffer by shaking for 1 h. Then, 3 nM CD63 aptamer solution was added to the MNP solution at room temperature with continuous shaking on a decoloring shaker for 2 h. The mixed solution was separated by a magnetic separation rack, and the supernatant was removed. The sediment was washed 3 times with PBS and resuspended in 20 μL of PBS. The obtained MNRs were stored at 4 °C for further use.

FTIR was carried out to confirm the conjugation between MNPs and the CD63 aptamers. The agate mortar was wiped with alcohol and dried under an infrared lamp. Then, 100 μL of the obtained MNRs solution was separated by using a magnetic separation rack, and the supernatant was removed by a manual pipette. The residual MNR precipitate was mixed with potassium bromide in accordance with a mass ratio of 100:1 in the agate mortar mentioned above. The mixture was then ground into flakes for further measurement.

DLS was used to measure the average size of MNPs and MNRs. Before starting the test, 10 μL of Milli-Q water was added to a disposable cuvette and used to calibrate the instrument as a blank sample. Ten microliters of MNP (2 mg/mL) solution and MNR solution, as prepared above, were transferred to the bottom of the disposable cuvette at 25 °C and then measured by a DLS instrument (30 sets of data each time for three cycles within five seconds).

TEM was performed to characterize the morphology of the MNPs, MNRs and MNRs-TDE conjugates. First, we prepared samples consisting of 30 μL of MNP solution (2 mg/mL), 30 μL of MNR solution (as prepared above), and a mixture of 30 μL of MNRs incubated with 30 μL of TDEs. The prepared samples were dropped on a sample-loading copper net and placed for 20 min. Next, the floating liquid was removed. After dyeing the samples on the copper net at room temperature for 5 min, the copper net was washed three times with Milli-Q water and observed by TEM.

To optimize the ratio between CD63 aptamers bound on the MNPs, FAM fluorophore-modified CD63 aptamers (CD63-FAM) at different concentrations (0.2, 0.5, 1, 2, 3, 4, and 5 μM) were prepared and measured by fluorescence spectrometry and then incubated with 20 μL of surface carboxyl-modified MNPs (2 mg/mL) to form MNRs^42^. The unreacted CD63-FAM on the supernatant was separated by a magnetic separation rack for half an hour, and then the supernatant was measured by a fluorescent spectrometer. The fluorescence intensity of the CD63 aptamer coupled to Fe_3_O_4_ MNPs was acquired by calculating the difference in the fluorescence intensity between CD63-FAM and the supernatant.

### Design and fabrication of microfluidic chip

The PDMS layer and curving agent were mixed thoroughly (10:1 by mass), poured on a mold, and cured in an oven at 80 °C for 30 min. The mold was fabricated from SU8-3025 negative photoresist (120 μm in depth) on a Si wafer. The PDMS layer (20 mM wide, 40 mM long) was peeled from the mold and trimmed after cooling.

The aldehyde group-modified glass slide and PDMS layer were bonded together by a YZD08-2CO_2_ plasma cleaner for 20 s under the protection of Eppendorf (EP) tube caps on the glass slide surface. All liquids in the experiment were accurately injected at constant and optimized flow rates by a programmable syringe pump.

When used for TDE detection, each microfluidic chip was incubated with 20 μL of DNA TSPs in an oven at 37 °C overnight^43^. After rinsing with 20 μL of PBS, an equal volume of SA (200 μg/mL) was linked with biotinylated DNA TSPs at 37 °C for 30 min. Then, 20 μL of biotin-CD9 antibody (50 μg/mL) was linked with SA by pumping it at 37 °C for 1 h to form a DNA TSP/SA/biotin-CD9 antibody structure to capture TDEs.

### Construction and optimization of the experimental conditions of the μFMS for TDE detection

The μFMS mainly consists of a microfluidic chip, PTFE tube, microsyringe pump and magnetic detector (Fig. S4). The PTFE tube was connected to the microfluidic chip and the magnetic detector. The microsyringe pump was used according to a program to drive the process. The magnetic detector was made of induction coils wrapped around a PTFE tube, differential and lock-in circuit (DLC), power supply and multimeter (Fig. S4A). The induction coils were used to excite the magnetic signal produced from MNP samples. The DLC was used to eliminate the spatial background noise and amplify the signal of MNPs (Fig. S4B).

When used for TDE detection, 20 μL of TDEs secreted from U251 cell lines and 20 μL of MNRs (2 mg/mL) were pumped into the microfluidic channels at room temperature and incubated with immobilized capture structures based on DNA TSPs for 1 h. The unreacted MNRs were rinsed with 20 μL of PBS buffer, collected in an EP tube at the outlet of the microfluidic chip and detected by a magnetic detector.

To test the effectiveness of the serpentine chip, two experiments were designed using a DNA TSP-modified serpentine chip and a flat chip. For both experimental groups, 1.98 × 10^5^ particle/mL TDEs from U251 cell lines were captured and incubated with MNRs in the serpentine chip and flat chip for 1 h at room temperature, and then the unreacted MNRs were detected as above.

To prove the effectiveness of DNA TSPs, two experiments including double-stranded DNA (dsDNA) and DNA TSPs as the capture scaffold immobilized on the aldehyde-modified glass slide were designed to recognize TDEs. For both experimental groups, 1.98 × 10^5^ particle/mL TDEs from U251 cell lines were detected in the μFMS as above.

To establish optimal conditions for TDE detection by the μFMS, the optimal incubation time was studied by incubating 1.98 × 10^5^ particle/mL TDEs from the U251 cell line with MNRs for 10, 30, 60, 90, and 120 min. The optimal flow rate was confirmed by using flow rates of 10, 15, 20 and 30 μL per min.

### Analytical performance study of the μFMS

For the sensitivity research of the μFMS, 20 μL of TDEs from U251 cell lines with different concentrations (from 1.98 × 10^3^ to 1.98 × 10^7^ particle/mL) and 20 μL of MNRs were pumped into the microfluidic chips to form TDE-MNR conjugates. The unreacted MNRs were rinsed with 20 μL of PBS and detected through μFMS as described above.

For the specificity research of the μFMS, CD9 antibody and three nonspecific antibodies (PSA antibody, BSA antibody and D-dimer antibody) with a concentration of 50 μg/mL were connected on SA, forming the DNA TSPs/SA/biotin–antibody structure. PBS solution without antibody was used as a control. Then, 20 μL TDEs from U251 cell lines with 1.98 × 10^5^ particle/mL and 20 μL MNRs (2 mg/mL) were pumped into the microfluidic chip. The unreacted MNRs were rinsed with 20 μL of PBS and detected with the μFMS as described above.

### Study on the clinical utility of the μFMS in simulated serum

To investigate the ability of our μFMS to detect exosomes in complex biological samples, 10 μL of 1.98 × 105 particles/mL TDEs from U251 cell lines and 4 μL of 1.98 × 105 particles/mL TDEs were added to 10 μL of FBS and 16 μL of FBS, respectively, to prepare 20 μL mixed samples containing 50 and 80% FBS. Then, 20 μL of simulated samples and 20 μL of MNRs (2 mg/mL) were pumped into the microfluidic chip for mixing. Then, the unreacted MNRs were rinsed with 20 μL of PBS and detected through μFMS as described above.

To further prove the feasibility of the fabricated μFMS for clinical samples, 2 μL, 1 μL and 0.4 μL TDEs (1.98 × 10^6^ particle/mL) isolated from the culture medium of U251 cells were added to 18 μL, 19 μL and 19.6 μL of FBS at final concentrations of 1.98 × 10^5^ particles/mL, 9.9 × 10^4^ particles/mL and 3.96 × 10^4^ particles/mL, respectively. The recovery rate was calculated using the standardization method.

## Results and discussion

### Working principle of the μFMS

**Fig. 1:**
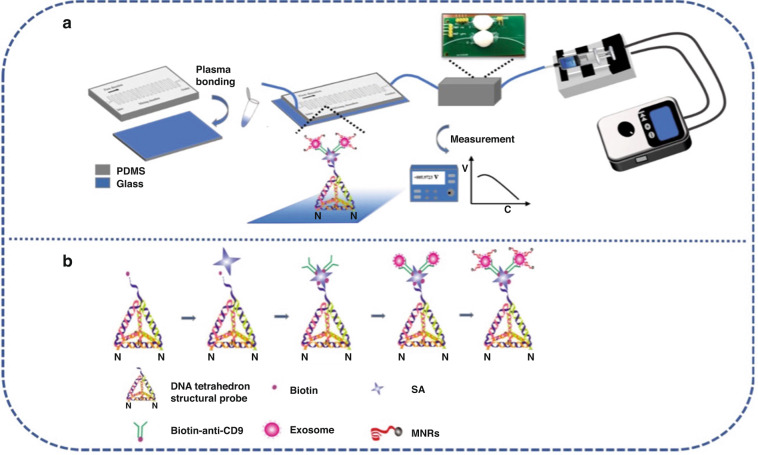
Working principle of the constructed μFMS for on-chip TDE capture and detection. **a** DNA TSPs immobilized on aldehyde-functionalized glass slides via covalent coupling between amine and aldehyde groups. **b** The sandwich structure among DNA TSPs/SA/biotin-anti CD9 capture structure

The TDEs were detected by the μFMS through a sandwich-like immunoassay. DNA TSPs were immobilized on aldehyde-functionalized glass slides via covalent coupling between amine and aldehyde groups^44^ (Fig. 1a). SA and biotin–anti-CD9 were injected into the microchannel sequentially to form the DNA TSPs/SA/biotin–anti-CD9 structure. The TDE and MNR solutions were pumped into the microfluidic chip via the inlet. While flowing through the serpentine mixing microchannel, the two solutions mixed and reacted. Then, the sandwich structure among the DNA TSPs/SA/biotin–anti-CD9 capture structure, TDEs and MNRs was formed (Fig. 1b). After that, PBS was used to rinse the MNRs, which were then collected into Eppendorf tubes. As the magnetic sensing platform we used is better at measuring volumetric-based magnetic signals generated from the MNRs than on the chip, we measured both the voltage output signal of the total MNRs (Voltage _total MNRs_) and the rinsed MNRs (Voltage _MNRs rinsed_) with the magnetometer magnetic detector. The voltage output signal of the MNRs with TDEs captured on chip (Voltage _MNRs on chip_) was acquired by calculating the difference between Voltage _total MNRs_ and Voltage _MNRs rinsed._

### Characterization of extracted TDEs

**Fig. 2:**
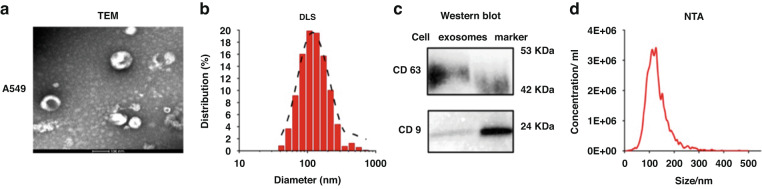
Characterization and Optimization of DNA TSPs and MNRs. **a** TEM of TDEs from U251 cell lines. **b** DLS of the particle diameter distribution of TDEs from U251 cell lines. **c** WB of CD63 and CD9 expressed on TDEs from U251 cell lines. **d** NTA of the sizes and concentration of TDEs from U251 cell lines

TEM was performed to observe the morphology of TDEs extracted from U251 cell lines. It was shown that the TDEs possess a typical cup-shaped membrane structure with an average size of 100 ~ 150 nm (Fig. 2a), which is consistent with a previous report^45^. DLS results showed that the average size of the TDEs was at the peak of 131 nm (Fig. 2b), confirming the presence of TDEs in our prepared samples. Clear bands for both CD63 and CD9 marker proteins were shown by WB (Fig. 2c), which indicated that CD63 and CD9 were expressed on the surface of TDEs. The size distribution and concentration of TDEs analyzed by NTA revealed that the concentration of TDEs extracted from U251 cell lines was 1.98 × 10^11^ particles/mL with an average size of 131 nm (Fig. 2d), which was consistent with the DLS results, confirming the presence of TDEs in our prepared samples and that they can be used for further study.

### Characterization and optimization of DNA TSPs and MNRs

**Fig. 3:**
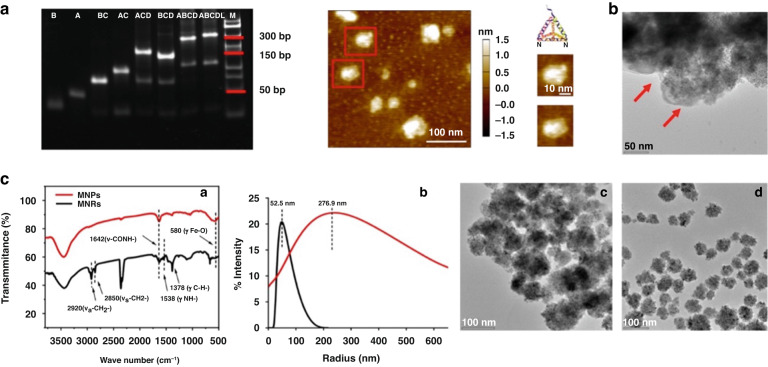
Characterization and Optimization of DNA TSPs and MNRs. **a** Gel electrophoresis image of different DNA strands and AFM results of DNA TSPs. **b** TEM image of the MNRs-TDEs complex; the scale bar is 50 nm. **c** FTIR spectra of MNPs (red) and MNRs (black); DLS results of MNPs and MNRs; TEM image of MNPs and MNRs, scale bar is 100 nm

SDS‒PAGE analysis was conducted to demonstrate the DNA tetrahedron performed by PCR. As shown in Fig. 3a (left), compared with images of two single-stranded DNAs (A, B), two double-stranded DNAs (AB, CD), two triple-stranded DNAs (ABC, BCD), one four-stranded DNA (ABCD), and one five-stranded DNA (ABCDL), the gel electrophoresis images indicated that DNA TSPs moved more slowly, which confirmed the successful formation of the DNA TSPs. The surface morphology of DNA TSPs is shown in Fig. 3a (right) by AFM. The triangular shape in the AFM image indicated that the DNA TSPs were assembled successfully.

The feasibility of combining MNRs and TDEs was confirmed by TEM. As shown in Fig. 3b, TDEs with typical phospholipid bilayer membrane structures (indicated by red arrows) were observed on the surface of the MNRs, indicating the successful capture of TDEs by the MNRs.

The binding of the aptamer on the MNPs depends on the dehydration condensation between carboxyl groups on MNPs and amino groups of the CD63 aptamer. TEM, FTIR spectra and DLS observations were used to confirm the conjugation between MNPs and the CD63 aptamers. As shown in Fig. 3c(a), two peaks at 580 cm^−1^ (Fe-O) and 1642 cm^−1^ (C = O) were observed in the FTIR spectra of the MNPs (black). After incubation with CD63 aptamers, four more peaks at 1378 cm^−1^ (C-N), 1538 cm^−1^ (N-H), 2850 cm^−1^ and 2920 cm^−1^ (CH2 in alkyl chains) were clearly observed in the FTIR spectra of MNRs (black). As shown in Fig. 3c(b), the size change between MNPs and MNRs revealed by DLS showed that the MNRs had a larger diameter (276.9 nm) than monodisperse MNPs (52.5 nm). The morphology shown by TEM indicated that the MNRs in Fig. 3c(c) underwent a slight aggregation, while the MNPs in Fig. 3c(d) showed good single dispersion. All the results above indicated that the MNP surface could be successfully coupled with the CD63 aptamer.

### Construction of the μFMS and optimization of the experimental conditions for TDE detection

The purpose of the microfluidic chip was to fix TSPs and capture TDEs. The size of the PDMS serpentine structure was 500 μm wide and 120 μm deep (Fig. S1), providing a large surface area compared with the straight structure (flat chip) (Fig. S2), which can enhance the capture efficiency of TDEs. Moreover, amino-modified DNA TSPs were easily fixed on aldehyde-functionalized glass slides by covalent coupling between amine and aldehyde groups.

The performance of the μFMS was calibrated by a concentration gradient of magnetic nanoparticle solutions (Fig. S5). The results showed that the generated voltage signal has a good linear correlation with the concentration of standard magnetic nanoparticles, and the linear regression equation was expressed as Y = 0.2091X + 0.00283 (R^2^ = 0.9997), which proved that the constructed μFMS system could quantitatively detect TDEs captured by MNRs.

The effectiveness of serpentine chips in μFMS for TDE detection was confirmed by using DNA TSP-modified serpentine chips and flat chips. The serpentine-chip group had a higher voltage signal than the flat-chip group, which means that more TDEs were captured on the chip (Fig. S6A).

The effectiveness of the DNA TSP-based scaffold for TDE detection was verified using a microfluidic chip made of an aldehyde-modified glass slide substrate immobilized by double-stranded DNA (dsDNA) and TSP DNA. The results showed that the DNA TSP group had a higher voltage signal than the dsDNA group, indicating that DNA TSPs had higher capture effectiveness (Fig. S6B).

The incubation time between TDEs and MNRs would affect the analysis process. A short incubation time would cause the TDEs to fail to bind to the MNRs completely. A long incubation time may aggravate the nonspecific absorption of the aptamers to the MNPs^46^. The flow rate in the μFMS would impact the binding efficiency between TDEs and MNRs. Low flow rates will increase sample processing time; however, high flow rates will cause inadequate incubation between TDEs and MNRs. To achieve higher assay performance for TDEs by μFMS, the incubation time and the flow rate were optimized.

**Fig. 4:**
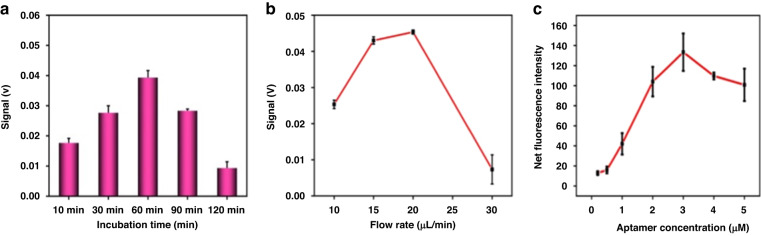
Optimization of the assay parameters. **a** Incubation time between TDEs and MNRs in the μFMS. **b** Flow rate in the μFMS. The error bar indicates the standard deviation of measurements (*n* = 3). **c** The relationship between the fluorescence intensity of CD63-FAM-MNPs and FAM-modified aptamer at different concentrations

As shown in Fig. 4a, with incubation times between TDEs and MNRs of 10, 30, 60, 90, and 120 min, 60 min could achieve the highest SNR (signal-to-noise ratio), which means the highest TDE capture efficiency on the microfluidic chip. Hence, 60 min was chosen as the optimal incubation time.

As shown in Fig. 4b, under the optimal incubation time (60 min), as the flow rate increased, the SNR signal increased, reached the highest value and began to decrease. The highest SNR achieved at a flow rate of 20 μL/min means that the highest capture efficiency for TDEs could be acquired. Therefore, 20 μL/min was chosen as the optimal flow rate.

Gradient experiments were designed to optimize the CD63 aptamer concentration bound on the MNPs. As shown in Fig. 4c, when the CD63 aptamer concentrations increased from 0.05 μM to 3 μM, the fluorescence intensity of CD63-FAM-MNPs gradually increased and became stable at a concentration of 3 μM, which indicated that the CD63 aptamer concentration loaded on the surface of MNPs had reached saturation. Therefore, 3 μM was chosen as the optimal aptamer concentration for this experiment.

### Analytical performance of the μFMS for TDEs

**Fig. 5:**
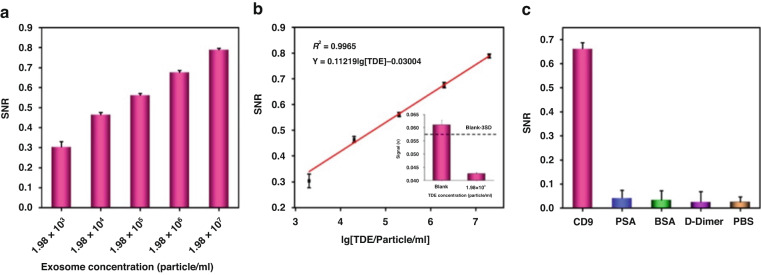
Analytical performance of the constructed μFMS for on-chip TDE capture and detection. **a** SNR of TDEs with different concentrations from U251 cell lines. **b** Curve fitting between SNR and the logarithm of the concentration of TDEs from U251 cell lines. **c** The specificity analysis of the μFMS. Error bars represent the SD of the SNR from three independent measurements

Once the experimental conditions of the μFMS for TDE detection were optimized, the isolated TDEs from U251 cell lines with different concentrations in PBS solution were analyzed on the microfluidic chip. The SNR of the MNRs increased as the TDE concentration increased from 1.98 × 10^3^ to 1.98 × 10^7^ particles/mL (Fig. 5a). The curve fitting between the SNR of MNRs and the logarithm of the concentration of TDEs reveals a good linear relationship (Fig. 5b). The correlation equation was expressed as SNR = 0.11219lg[TDE]-0.03004 (R^2^ = 0.9965). As the SNR at a concentration of 1.98 × 10^3^ particles/mL was obviously lower than the threshold, which was equal to the blank signal plus three standard deviations (3 SD), a limit of detection (LOD) of 1.98 × 10^3^ particles/mL was obtained (Fig. 5b, inset).

Our μFMS method and other reported microfluidic platforms for TDE detection is shown in Table 1. Compared with most of the other works, our μFMS had the advantages of easy miniaturization, integration and low cost, which could meet the requirements of clinical POCT. Moreover, the combined use of MNPs, DNA TSPs and microfluidic chips with magnetic biosensors ensures that this method can achieve sensitive and rapid detection ability.

**Table 1:** TDEs detection and analysis in microfluidic platform

Analytical method	LOD (Particle /mL)	Volume (μL)	Linear range (Particle/mL)	Time	Ref.
Nano-IMEX platform	5 × 10^4^	2	10^3^-10^6^	within 3 h	^18^
A Microfluidic ExoSearch Chip	**/**	20	5 × 10^5^–1 × 10^7^	within 40 min	^38^
ExoPCD-chip	4.39 × 10^3^	30	7.61 × 10^4^–7.61 × 10^8^	with 3.5 h	^47^
Microfluidic Raman biochip	1.6 × 10^2^~1.6 x 10^9^	20	1.6 × 10^2^	with 3.5 h	^21^
Microfluidic-Electrochemical Aptasensor	1.0 × 10^6^	/	10^6^–10^9^	within 1 h	^24^
Magnetic-electrochemical exosome platform	3.96 × 10^5^	10	10^6^–10^10^	/	^23^
The μFMS	1.98 × 10^3^	20	1.98 × 10^3^–1.98 × 10^7^	about 1 h	This work

The results of the specificity research for μFMS are shown in Fig. 5c. The measured value of SNR using nonspecific antibodies was as low as that of the blank (PBS). However, the measured value of SNR using CD9 antibody was much higher than that of the blank. The high specificity of μFMS was mainly attributed to the specific binding between aptamers and antibodies with the surface proteins of TDEs.

### Detection of exosomes in complex biosamples

**Fig. 6:**
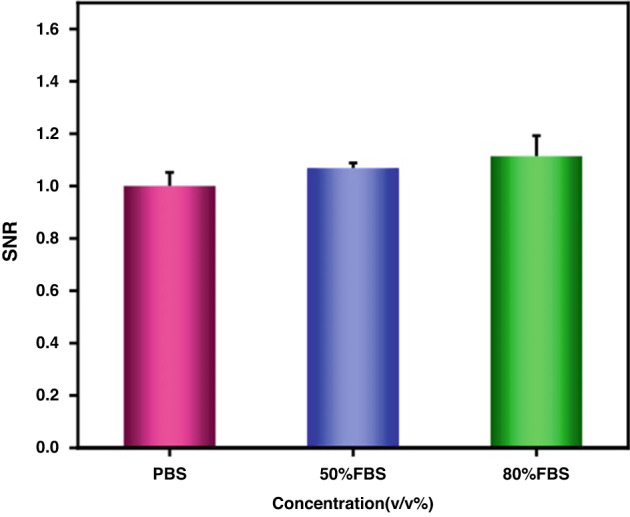
Detection of the TDEs from U251 cell lines in PBS and simulated serum samples

To verify the clinical utility of the μFMS for TDEs, the detection performance of our μFMS was evaluated by simulated clinical serum samples. There was no difference among the SNRs of TDEs extracted from U251 cell lines in PBS solution and simulated serum samples with 50% FBS and 80% FBS (Fig. 6), which means that our constructed μFMS could be a potential analytical method to detect TDEs from real clinical samples.

The recovery rate was also used to evaluate the feasibility of the fabricated μFMS for clinical samples. The acquired results were 100%, 106.83%, and 111.38%, respectively, which indicated that our μFMS could achieve good detection performance for TDEs in complex environments (Table 2).

**Table 2:** TDE detection in simulated clinical serum samples

No	Added particles/mL	Count (particles/ mL)	Recovery (%), *n* = 3
1	1.98 × 10^5^ particle/mL	1.98 × 10^5^ particle/mL	100%
2	9.9 × 10^4^ particle/mL	4.01 × 10^5^ particle/mL	106.83%
3	3.96 × 10^4^ particle/mL	4.09 × 10^5^ particle/mL	111.38%

## Conclusion

In this work, we developed a DNA tetrahedron-mediated μFMS for the on-chip detection of TDEs with high sensitivity and specificity. We designed and fabricated a microfluidic chip with PDMS as the upper layer and glass substrate as the lower layer. DNA tetrahedron nanoprobes were synthesized and modified on a glass substrate to capture TDEs. The microfluidic chip was combined with a magnetic detector containing induction coils and a differential-amplification circuit. After the feasibility of DNA tetrahedron nanoprobes and the microfluidic chip for measuring TDEs in μFM was verified, the detection performance of the μFMS was determined under the optimized conditions with a linear dynamic detection range of 1.98 × 10^3^–1.98 × 10^7^ particles/mL and a limit of detection of 1.98 × 10^3^ particle/mL. We proved that there was no significant difference in the detection results of TDEs between the simulated serum system and the PBS buffer. Compared with other strategies for TDE detection, such as optical or electrochemical methods, our μFMS showed great advantages in detection sensitivity and time. Therefore, the μFMS system we developed could provide an extraordinary option for noninvasive liquid biopsy and hold the potential to be a useful POCT tool.

### Supplementary information

text summary for supplementary information

Supporting Information Microfluidic Magnetic Detection System based on DNA Tetrahedron-Mediated immune-sandwich assay for rapid and sensitive detection of Tumor-derived Exosomes

## References

[1] Ferlay, J. et al. Cancer statistics for the year 2020: An overview. *Int. J. Cancer* **149**, 778–789 (2021). 10.1002/ijc.33588 33818764

[2] Roma-Rodrigues C, Fernandes AR, Baptista PV (2014). Exosome in Tumour Microenvironment: Overview of the Crosstalk between Normal and Cancer Cells. BioMed. Res. Int..

[3] Johnstone RM, Adam M, Hammond JR, Orr L, Turbide C (1987). Vesicle formation during reticulocyte maturation. Association of plasma membrane activities with released vesicles (exosomes). J. Biol. Chem..

[4] Valadi H (2007). Exosome-mediated transfer of mRNAs and microRNAs is a novel mechanism of genetic exchange between cells. Nat. Cell Biol..

[5] Jeppesen DK (2019). Reassessment of Exosome Composition. Cell.

[6] Vlassov AV, Magdaleno S, Setterquist R, Conrad R (2012). Exosomes: Current knowledge of their composition, biological functions, and diagnostic and therapeutic potentials. Biochimica et. Biophysica Acta (BBA) - Gen. Subj..

[7] Doyle L, Wang M (2019). Overview of Extracellular Vesicles, Their Origin, Composition, Purpose, and Methods for Exosome Isolation and Analysis. Cells.

[8] Thery C, Zitvogel L, Amigorena S (2002). Exosomes: composition, biogenesis and function. Nat. Rev. Immunol..

[9] Van Niel G, D’Angelo G, Raposo G (2018). Shedding light on the cell biology of extracellular vesicles. Nat. Rev. Mol. Cell Biol..

[10] Boyiadzis M, Whiteside TL (2017). The emerging roles of tumor-derived exosomes in hematological malignancies. Leukemia.

[11] Norouzi-Barough L (2020). Early diagnosis of breast and ovarian cancers by body fluids circulating tumor-derived exosomes. Cancer Cell Int..

[12] Chen IH (2017). Phosphoproteins in extracellular vesicles as candidate markers for breast cancer. Proc. Natl Acad. Sci..

[13] Cavallaro S (2019). Label-free surface protein profiling of extracellular vesicles by an electrokinetic sensor. ACS Sens..

[14] Oksvold MP, Neurauter A, Pedersen KW (2015). Magnetic bead-based isolation of exosomes. Methods Mol. Biol..

[15] Gao ML, He F, Yin BC, Ye BC (2019). A dual signal amplification method for exosome detection based on DNA dendrimer self-assembly. Analyst.

[16] He F, Liu H, Guo X, Yin B-C, Ye B-C (2017). Direct exosome quantification via bivalent-cholesterol-labeled DNA anchor for signal amplification. Anal. Chem..

[17] Liang LG (2017). An integrated double-filtration microfluidic device for isolation, enrichment and quantification of urinary extracellular vesicles for detection of bladder cancer. Sci. Rep..

[18] Zhang P, He M, Zeng Y (2016). Ultrasensitive microfluidic analysis of circulating exosomes using a nanostructured graphene oxide/polydopamine coating. Lab Chip.

[19] Liu C (2018). Sensitive detection of exosomal proteins via a compact surface plasmon resonance biosensor for cancer diagnosis. ACS Sens..

[20] Wang Z (2018). Screening and multiple detection of cancer exosomes using an SERS-based method. Nanoscale.

[21] Wang Y (2020). Microfluidic Raman biochip detection of exosomes: a promising tool for prostate cancer diagnosis. Lab Chip.

[22] Zong S (2016). Facile detection of tumor-derived exosomes using magnetic nanobeads and SERS nanoprobes. Anal. Methods.

[23] Wang S (2017). Aptasensor with Expanded Nucleotide Using DNA Nanotetrahedra for Electrochemical Detection of Cancerous Exosomes. ACS Nano.

[24] Zhou Q (2016). Development of an aptasensor for electrochemical detection of exosomes. Methods.

[25] Park, J. et al. An integrated magneto-electrochemical device for the rapid profiling of tumour extracellular vesicles from blood plasma. *Nat. Biomed. Eng.* **5**, 678–689 (2021). 10.1038/s41551-021-00752-7 PMC8437135 34183802

[26] Issadore D (2014). Magnetic sensing technology for molecular analyses. Lab Chip.

[27] Huber DL (2005). Synthesis, properties, and applications of iron nanoparticles. Small.

[28] Pastucha M, Farka Z, Lacina K, Mikusova Z, Skladal P (2019). Magnetic nanoparticles for smart electrochemical immunoassays: a review on recent developments. Mikrochim Acta.

[29] Wu K (2020). Magnetic Particle Spectroscopy: A Short Review of Applications Using Magnetic Nanoparticles. ACS Appl. Nano Mater..

[30] Yüce M, Ullah N, Budak H (2015). Trends in aptamer selection methods and applications. Analyst.

[31] Wang W (2016). A magnetic nanoparticles relaxation sensor for protein-protein interaction detection at ultra-low magnetic field. Biosens. Bioelectron..

[32] Wu K (2017). Portable GMR Handheld Platform for the Detection of Influenza A Virus. ACS Sens..

[33] Shao H (2012). Protein typing of circulating microvesicles allows real-time monitoring of glioblastoma therapy. Nat. Med..

[34] Issadore D (2012). Ultrasensitive clinical enumeration of rare cells ex vivo using a micro-hall detector. Sci. Transl. Med..

[35] Sasayama, T., Yoshida, T., Enpuku, K. Two-dimensional magnetic nanoparticle imaging using multiple magnetic sensors based on amplitude modulation. *J. Magnet. Magnetic Mater.* **505**, 166765 (2020).

[36] Murzin, D. et al. Ultrasensitive Magnetic Field Sensors for Biomedical Applications. *Sensors (Basel)* **20**,1569 (2020). 10.3390/s20061569 PMC7146409 32168981

[37] Contreras-Naranjo JC, Wu H-J, Ugaz VM (2017). Microfluidics for exosome isolation and analysis: enabling liquid biopsy for personalized medicine. Lab a Chip.

[38] Zhao Z, Yang Y, Zeng Y, He M (2016). A microfluidic ExoSearch chip for multiplexed exosome detection towards blood-based ovarian cancer diagnosis. Lab a Chip.

[39] Fang, S. et al. Clinical application of a microfluidic chip for immunocapture and quantification of circulating exosomes to assist breast cancer diagnosis and molecular classification. *PLoS One* **12**, e0175050 (2017). 10.1371/journal.pone.0175050 PMC5378374 28369094

[40] Chen, X. et al. Ultrasensitive electrochemical detection of prostate-specific antigen by using antibodies anchored on a DNA nanostructural scaffold. *Anal. Chem.* **86**, 7337–7342 (2014). 10.1021/ac500054x 24965743

[41] Feng, D. et al. DNA tetrahedron-mediated immune-sandwich assay for rapid and sensitive detection of PSA through a microfluidic electrochemical detection system. *Microsyst. Nanoeng.* **7**, 33 (2021). 10.1038/s41378-021-00258-x PMC8433179 34567747

[42] Wang Y (2020). SERS-based immunocapture and detection of pathogenic bacteria using a boronic acid-functionalized polydopamine-coated Au@Ag nanoprobe. Mikrochim. Acta..

[43] Li Z (2014). DNA nanostructure-based universal microarray platform for high-efficiency multiplex bioanalysis in biofluids. ACS Appl. Mater. Interfaces.

[44] Lin M (2016). Electrochemical detection of nucleic acids, proteins, small molecules and cells using a DNA-nanostructure-based universal biosensing platform. Nat. Protoc..

[45] Sunkara V, Woo HK, Cho YK (2016). Emerging techniques in the isolation and characterization of extracellular vesicles and their roles in cancer diagnostics and prognostics. Analyst.

[46] Yu X (2019). An aptamer-based new method for competitive fluorescence detection of exosomes. Nanoscale.

[47] Xu H, Liao C, Zuo P, Liu Z, Ye BC (2018). Magnetic-Based Microfluidic Device for On-Chip Isolation and Detection of Tumor-Derived Exosomes. Anal. Chem..

