# Supplementary material for: Microfluidic magnetic detection system combined with a DNA framework-mediated immune-sandwich assay for rapid and sensitive detection of tumor-derived exosomes
Source: Microsyst Nanoeng. 2023 Nov 7;9:139. doi: 10.1038/s41378-023-00617-w (PMC10630345; doi:10.1038/s41378-023-00617-w)
Supplement: Supplementary file 1 — text summary for supplementary information [file 41378_2023_617_MOESM1_ESM.docx]

The supporting information file includes the CAD diagram and the actual drawing of microfluidic chip, the physical diagram of μFMS, the schematic of magnetic detector, the description of the principle and the calibration curve of the constructed μFMS, and the base sequences used in the experiment.
