# Supplementary material for: Microfluidic magnetic detection system combined with a DNA framework-mediated immune-sandwich assay for rapid and sensitive detection of tumor-derived exosomes
Source: Microsyst Nanoeng. 2023 Nov 7;9:139. doi: 10.1038/s41378-023-00617-w (PMC10630345; doi:10.1038/s41378-023-00617-w)
Supplement: Supplementary file 2 — Supporting Information Microfluidic Magnetic Detection System based on DNA Tetrahedron-Mediated immune-sandwich assay for rapid and sensitive detection of Tumor-derived Exosomes [file 41378_2023_617_MOESM2_ESM.docx]

**Supporting Information**

**Microfluidic Magnetic Detection System based on DNA Tetrahedron-Mediated immune-sandwich assay for rapid and sensitive detection of Tumor-derived Exosomes**

Qiuling Qian ^a,b,c1^,Yutong Wei ^a,c,e1^, Yi Xu^a,b^, Mengmeng Zheng ^b,d^, Chenguang Wang ^b,c^ , Shulin Zhang ^a^, Xiaoming Xie ^a*^, Chaofeng Ye ^e*^, Xianqiang Mi ^a,b,c,f,g*^

^a^State Key Laboratory of Functional Materials for Informatics, Shanghai Institute of Microsystem and Information Technology, Chinese Academy of Sciences, Shanghai 200050, China

^b^Shanghai Advanced Research Institute, Chinese Academy of Sciences, Shanghai,201210, China

^c^University of Chinese Academy of Sciences, Beijing 100049, China

^d^School of Life Sciences, Shanghai University, Shanghai, 200444, China

^e^School of Information Science and Technology, Shanghai Tech University, Shanghai 201210, China.

^f^Key Laboratory of Systems Health Science of Zhejiang Province, Hangzhou Institute for Advanced Study, University of Chinese Academy of Sciences, Chinese Academy of Sciences, 310024 Hangzhou, China

^g^Research Center for Sensing Materials and Devices Zhejiang Lab, Hangzhou, Zhejiang,311121, China

*Corresponding author.

^1^ Equal contributing authors.

*E-mail address:* [mixq@mail.sim.ac.cn](mailto:mixq@mail.sim.ac.cn) (Xianqiang Mi)

[yechf@shanghaitech.edu.cn](mailto:yechf@shanghaitech.edu.cn) (Chaofeng Ye)

^1^ Equal contributing authors.

**Supplementary Figure**


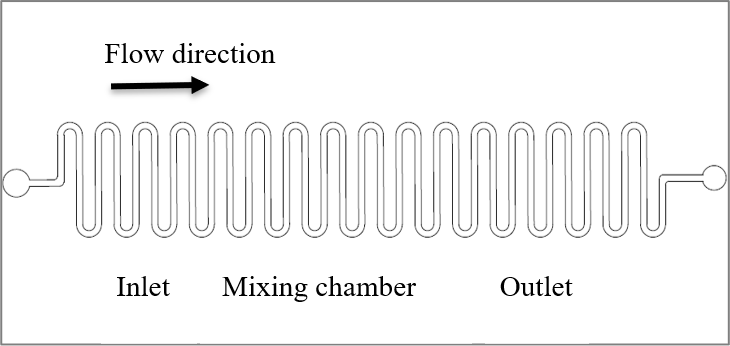


**Fig.S1.**CAD diagram of the Serpentine Channel of Microfluidic chip

**
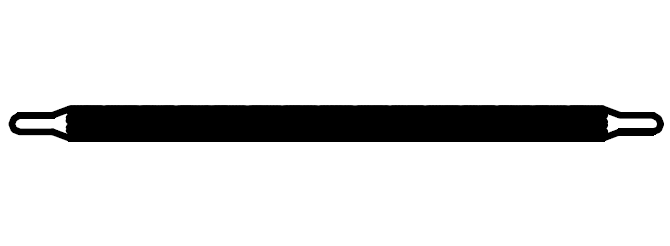
**

**Fig.S2.** CAD diagram of the Straight Channel of Microfluidic chip

**Figure S3.** The Actual installation drawing of the constructed microfluidic chip.


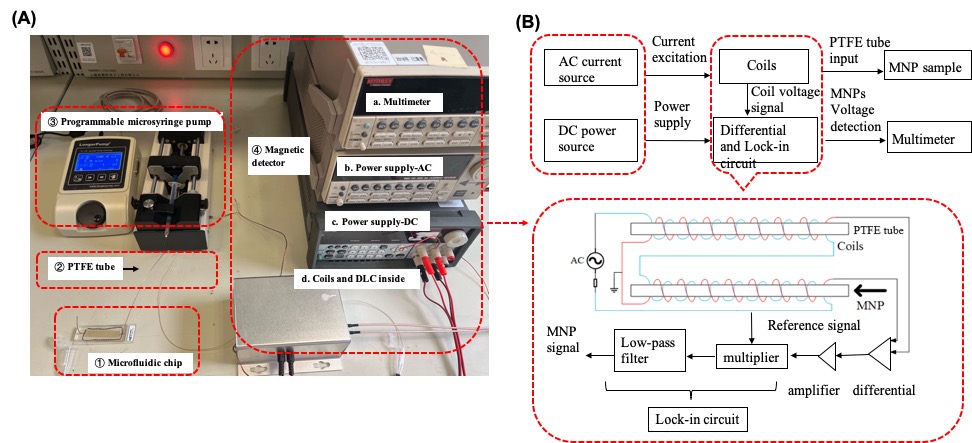


**Figure S4.** (A) the physical diagrams of μFMS. (B) the schematic of magnetic detector consists of induction coils wrapped around PTFE tube, differential amplification circuit (DAC), power supply and multimeter.

**Principle of the constructed uFMS:**

In the microfluidic magnetic bio-detection system (μFMS), the analog quantity of biological signal detection is converted into a weak electrical signal after passing through the sensor. In order to better measure these weak signals, it is generally amplified. The usual method of directly coupling the amplifier circuit to drive the load is accompanied by background noise or zero drift. This is caused by electromagnetic interference in the environment or different characteristics of the circuit components themselves.

The differential amplification circuit uses a transistor-integrated circuit with the same characteristics, which eliminates the electromagnetic interference inherent in the environment and the interference caused by the different characteristics of the components themselves. Generally, we input the signal that needs to be amplified as a differential-mode signal, and the influence of environmental factors such as temperature on the circuit as a common-mode signal input, so as to amplify the differential-mode signal and suppress the common-mode signal to achieve the purpose of amplification. Due to the symmetry of the differential circuit, when the signal connected to the two input terminals is equal in size and the same polarity, it becomes a common-mode signal, and when the signal connected to the two input terminals is equal in size and polarity is opposite, it is called a differential mode signal. Therefore, the differential amplification circuit has a strong suppression effect on the common-mode signal and amplification effect on the differential-mode signal, which can meet our ultimate purpose.


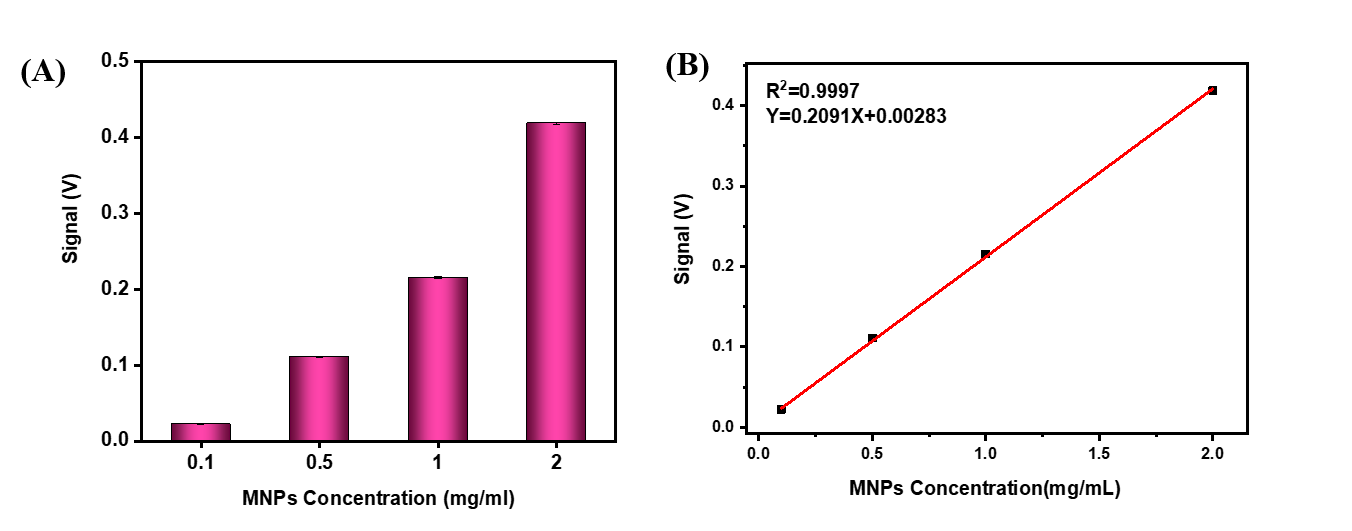


**Figure S5.** The calibration curve of the constructed μFMS; The concentration of standard magnetic nanoparticles flowing through the microfluidic chip channel has a good linear correlation with the output value of the generated voltage signal in μFMS

**
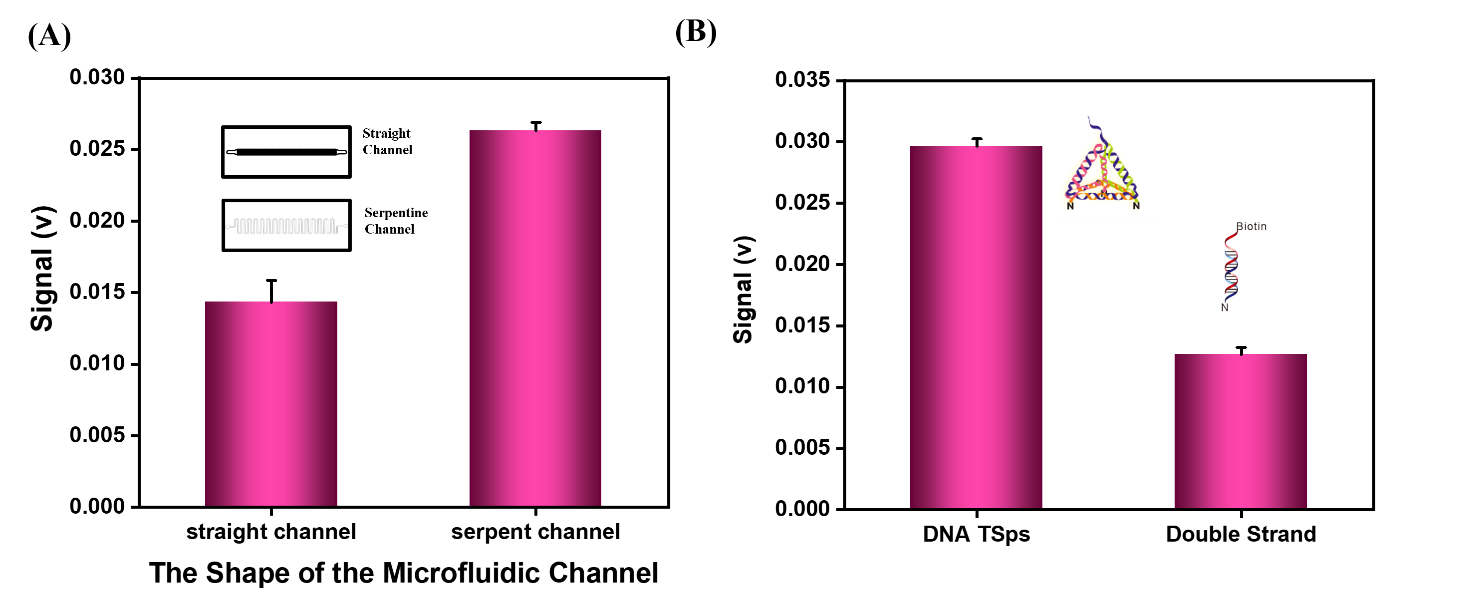
**

**Figure S6.** (A) the shape of the microfluidic channel. (B）the type of capture scaffold immobilized on the aldehyde functionalized glass slide.


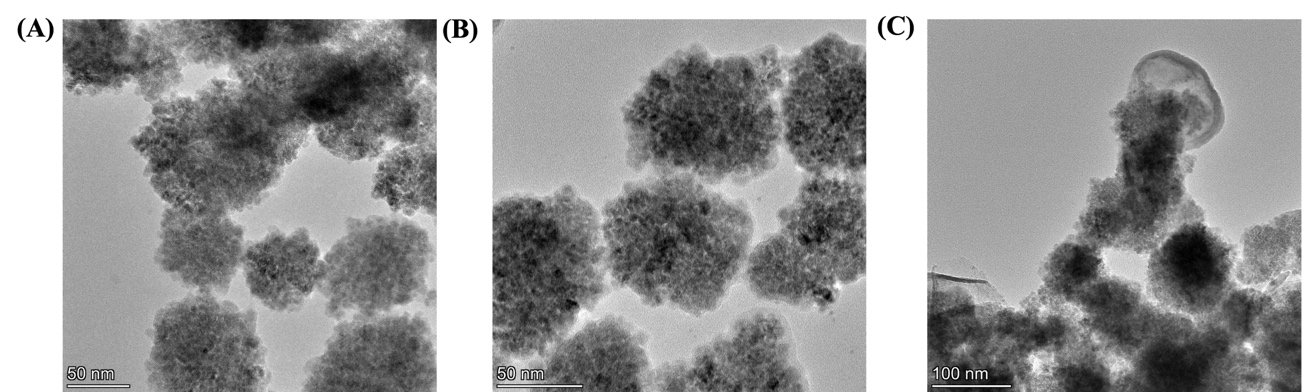


**
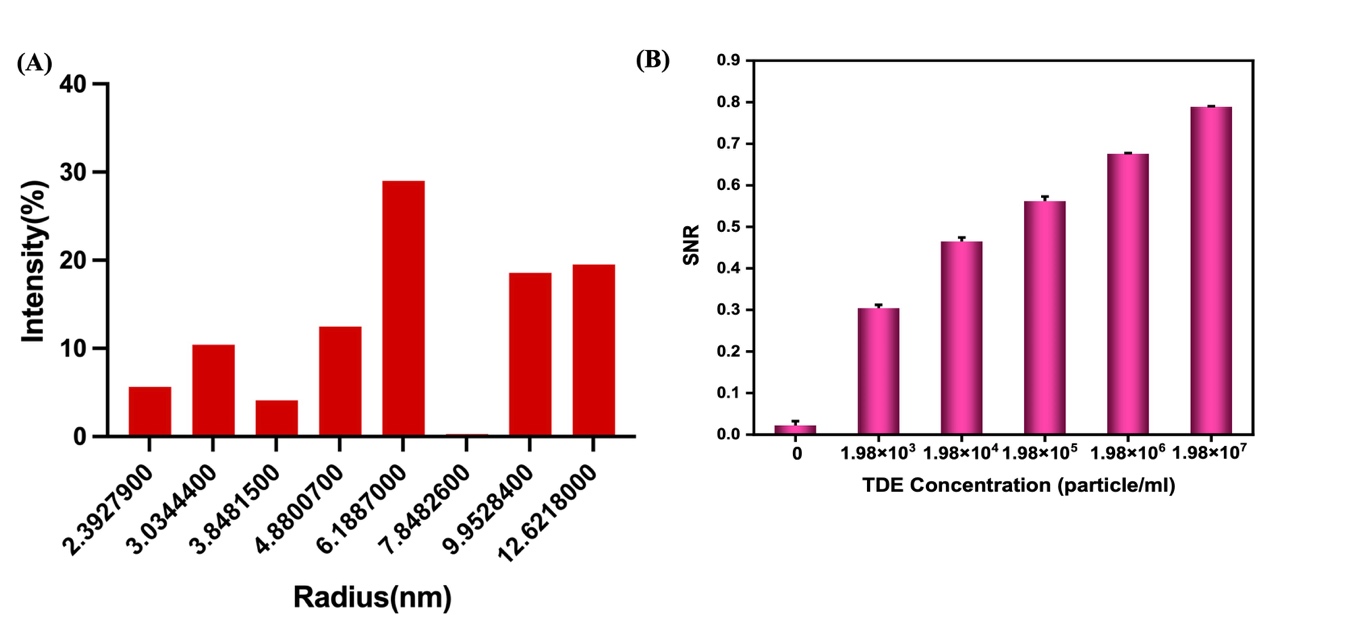
Figure S7：**TEM image of MNPs（A）,MNRs （B）and MNRs-TDEs complex (C), scale bar is 50nm，50nm and 100nm respectively.

**Figure S8.** The DLS characterization of the TSPs (A) and the voltage value change before and after TDEs capture(B).

**Supplementary Table**

| Name | Sequence（5’-3’） | |
| --- | --- | --- |
| A-probe | | ACATTCCTAAGTCTGAAACATTACAGCTTGCTACACGAGAAGAGCCGCCATAGTATTTTTTTTTTGTATCCAGTGGCTCA |
| B-probe | | NH_2_-C6-TATCACCAGGCAGTTGACAGTGTAGCAAGCTGTAATAGATGC GAGGGTCCAATAC |
| C-probe | | NH_2_-C6-TCAACTGCCTGGTGATAAAACGACACTACGTGGGAATCTACT ATGGCGGCTCTTC |
| D-probe | | NH_2_-C6-TTCAGACTTAGGAATGTGCTTCCCACGTAGTGTCGTTTGTATT GGACCCTCGCAT |
| NH_2_-CD63-aptamer | | NH_2_-CACCCCACCTCGCTCCCGTGACACTAATGCTA |
| Linker1  Linker2 | | Biotin-TGAGCCACTGGATAC  NH_2_-TTTTTTTTTTGTATCCAGTGGCTCA |

**Table S1.** The base sequences used in the experiment.
